# Supplementary figures and images for: Abnormal arachidonic acid metabolic network may reduce sperm motility via P38 MAPK
Source: Open Biol. 2019 Apr 24;9(4):180091. doi: 10.1098/rsob.180091 (PMC6501647; doi:10.1098/rsob.180091)

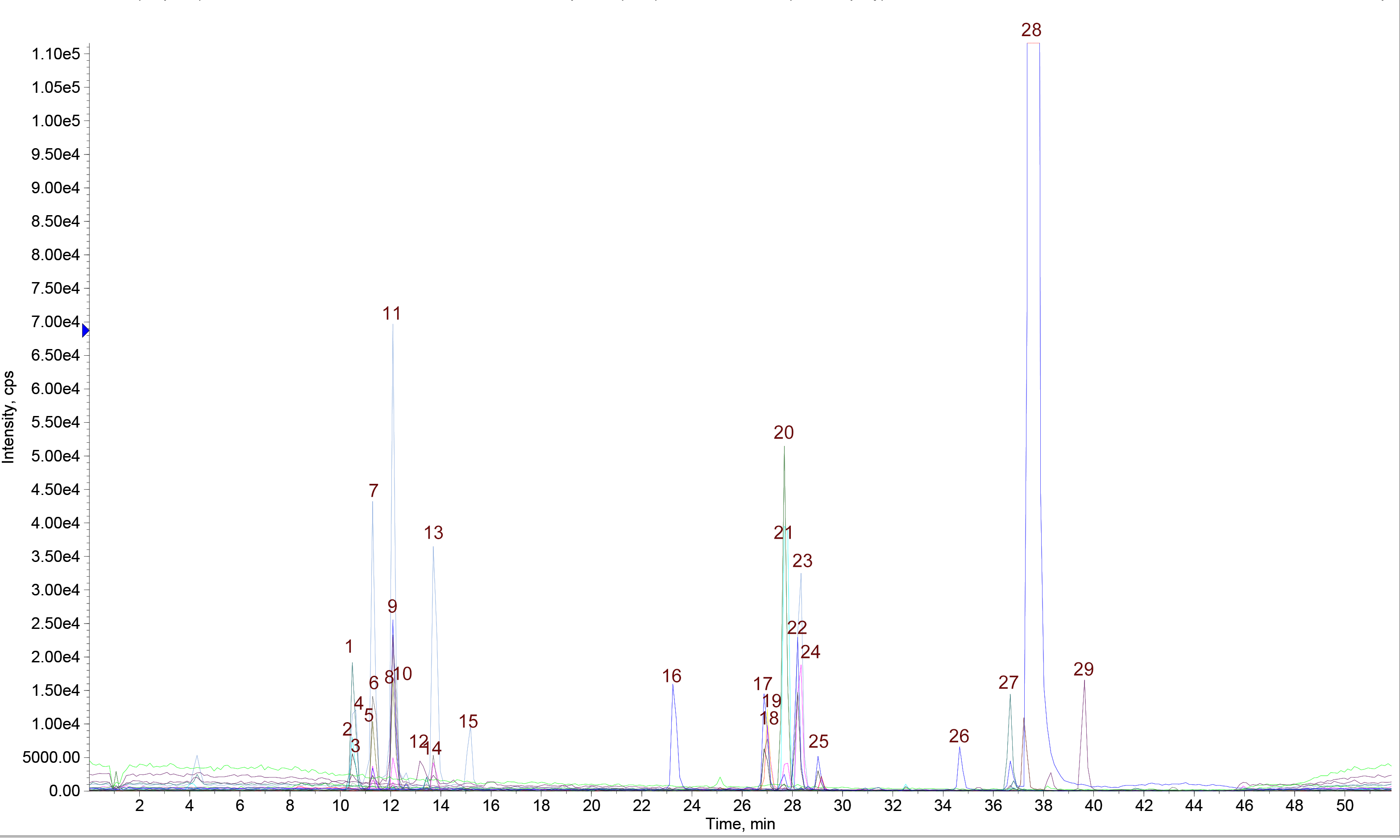

Supplement: Supplementary Fig. 1. Representative HPLC-ESI-MS/MS chromatogram of human seminal plasma [file rsob180091supp1.tif]
