# Supplementary material for: Abnormal arachidonic acid metabolic network may reduce sperm motility via P38 MAPK
Source: Open Biol. 2019 Apr 24;9(4):180091. doi: 10.1098/rsob.180091 (PMC6501647; doi:10.1098/rsob.180091)
Supplement: Supplementary Table 1 [file rsob180091supp2.doc]

**Open Biology**

**Abnormal arachidonic acid metabolic network may reduce sperm motility via P38 MAPK**

Lisha Yu^1^, Xiaojing Yang^1^, Bo Ma^1^, Hanjie Ying^2^, Xuejun Shang^3,***^ , Bingfang He^1,**^, Qi Zhang^1,*^

**Supplementary Table 1.** Characteristics of the study population.

| **Parameters** | **Healthy (n=33)** | **Asthenozoospermia (n=30)** | **P Value** |
| --- | --- | --- | --- |
| Age (year) | 31.23 ± 6.36 | 33.18 ± 5.73 | 0.3278 |
| BMI (kg·cm^-2^) | 23.39 ± 2.97 | 22.55 ± 3.16 | 0.5872 |
| Sperm concentration (×10^6^ ml^-1^) | 43.38 ± 28.72 | 41.96 ± 31.97 | 0.1928 |
| Sperm progressive motility (a+b, %) | 57.15 ± 14.67 | 39.87 ± 16.44 | < 0.001 |
| Straight-line velocity (μm·s^-1^) | 42.38 ± 7.42 | 28.56 ± 7.67 | < 0.001 |
| Curve-line velocity (μm·s^-1^) | 71.05 ± 14.11 | 52.97 ± 14.57 | < 0.001 |
| Average path velocity (μm·s^-1^) | 49.25 ± 9.94 | 34.18 ± 9.0 | < 0.001 |
| Duration of abstinence (day) | 4.01 ± 0.23 | 4.0 ± 0.28 | 1.2546 |
