# Supplementary material for: Abnormal arachidonic acid metabolic network may reduce sperm motility via P38 MAPK
Source: Open Biol. 2019 Apr 24;9(4):180091. doi: 10.1098/rsob.180091 (PMC6501647; doi:10.1098/rsob.180091)
Supplement: Supplementary Table 2 [file rsob180091supp3.doc]

**Open Biology**

**Abnormal arachidonic acid metabolic network may reduce sperm motility via P38 MAPK**

Lisha Yu^1^, Xiaojing Yang^1^, Bo Ma^1^, Hanjie Ying^2^, Xuejun Shang^3,***^ , Bingfang He^1,**^, Qi Zhang^1,*^

**Supplementary Table 2.** The specific precursor and product ions of the analytes in MRM mode by HPLC-ESI-MS/MS.

| **Compound** | **Q1 Mass (m/z)** | **Q3 Mass (m/z)** | **DP /V** | **CE /eV** |
| --- | --- | --- | --- | --- |
| Arachidonic acid | 303 | 259 | -100 | -19 |
| 5-HETE | 319 | 115 | -92 | -19 |
| 8-HETE | 319 | 155 | -92 | -22 |
| 9-HETE | 319 | 151 | -92 | -20 |
| 12-HETE | 319 | 179 | -92 | -20 |
| 20-HETE | 319 | 301 | -92 | -22 |
| 5(S)-HpETE | 335 | 317 | -61 | -7 |
| 5,6-EET | 319.3 | 191.4 | -76 | -16 |
| 8,9-EET | 319 | 123 | -68 | -20 |
| 11,12-EET | 319 | 167 | -68 | -18 |
| 14,15-EET | 319 | 219 | -68 | -15 |
| 5,6-DHET | 337.4 | 145 | -100 | -25 |
| 8,9-DHET | 337.4 | 127 | -100 | -29 |
| 11,12-DHET | 337.4 | 167 | -100 | -25 |
| 14,15-DHET | 337.4 | 207.1 | -100 | -25 |
| LTB_4_ | 335 | 195 | -130 | -22 |
| LTC_4_ | 624 | 272 | -120 | -33 |
| LTD_4_ | 495 | 177 | -100 | -27 |
| LTE_4_ | 438 | 333 | -102 | -25 |
| LXA_4_ | 351 | 115 | -100 | -19 |
| PGJ_2_ | 333 | 189 | -100 | -24 |
| Tetranor-PGEM | 327 | 309 | -50 | -16 |
| Tetranor-PGFM | 329 | 311 | -55 | -18 |
| 6-KetoPGF_1α_ | 369 | 163 | -90 | -35 |
| 13,14-dIHYDRO-15-ketoPGF_1α_ | 353 | 113 | -150 | -36 |
| 8-IsoPGF_2α_ | 353 | 193 | -137 | -34 |
| 11-DehydroTXB_2_ | 367 | 305 | -100 | -22 |
| 2,3-Dinor TXB_2_ | 341 | 123 | -100 | -23 |
| PGE_2_ | 351.3 | 315.3 | -90 | -16 |
| PGF_2α_ | 353 | 291 | -103 | -28 |
| PGD_2_ | 351 | 233 | -90 | -17 |
| TXB_2_ | 369 | 169 | -90 | -20 |
| 11-HETE | 319 | 167 | -92 | -22 |
| 15-HETE | 319 | 219 | -92 | -17 |
| 13,14-dIHYDRO-15-ketoPGD_2_ | 351 | 333 | -60 | -16 |
| 13,14-dIHYDRO-15-ketoPGE_2_ | 351 | 235 | -60 | -30 |
| 11β-PGF_2α_ | 353 | 309 | -72 | -27 |
| PGF_2β_ | 353 | 335 | -72 | -18 |
| 13,14-dIHYDRO-15-ketoPGF_2α_ | 353 | 291 | -72 | -29 |
| 12(S)-HpETE | 335.2 | 273.2 | -61 | -11 |
| 15(S)-HpETE | 335.2 | 113 | -61 | -14 |
| 9(S)-HpODE | 311 | 293 | -66 | -12 |
| 13(S)-HpODE | 311 | 113 | -66 | -22 |
| N-LTE_4_ | 480.3 | 351.1 | -102 | -24 |
| LTF_4_ | 567.3 | 171.1 | -100 | -34 |
| PGB_2_ | 333.2 | 234.8 | -100 | -26 |
