# Supplementary material for: Abnormal arachidonic acid metabolic network may reduce sperm motility via P38 MAPK
Source: Open Biol. 2019 Apr 24;9(4):180091. doi: 10.1098/rsob.180091 (PMC6501647; doi:10.1098/rsob.180091)
Supplement: Supplementary Table 3 [file rsob180091supp4.doc]

**Open Biology**

**Abnormal arachidonic acid metabolic network may reduce sperm motility via P38 MAPK**

Lisha Yu^1^, Xiaojing Yang^1^, Bo Ma^1^, Hanjie Ying^2^, Xuejun Shang^3,***^ , Bingfang He^1,**^, Qi Zhang^1,*^

**Supplementary Table 3.** The stability of QC samples.

| **IS** | **Peak area** |
| --- | --- |
| 1 | 3.2547E+05 |
| 2 | 3.4214E+05 |
| 3 | 3.2297E+05 |
| 4 | 2.9656E+05 |
| 5 | 2.9345E+05 |
| 6 | 3.8282E+05 |
| 7 | 3.7660E+05 |
| 8 | 3.7095E+05 |
| MEAN  SD  RSD | 3.39E+05  3.52E+04  10.39% |
