# Supplementary material for: Abnormal arachidonic acid metabolic network may reduce sperm motility via P38 MAPK
Source: Open Biol. 2019 Apr 24;9(4):180091. doi: 10.1098/rsob.180091 (PMC6501647; doi:10.1098/rsob.180091)
Supplement: Supplementary Table 4 [file rsob180091supp5.doc]

**Open Biology**

**Abnormal arachidonic acid metabolic network may reduce sperm motility via P38 MAPK**

Lisha Yu^1^, Xiaojing Yang^1^, Bo Ma^1^, Hanjie Ying^2^, Xuejun Shang^3,***^ , Bingfang He^1,**^, Qi Zhang^1,*^

**Supplementary Table 4.** Linear equations and correlation coefficients of representative AA metabolites.

| **Compound** | **Linear equation** | **Correlation coefficient** | **Linear range**  **(ng/mL)** |
| --- | --- | --- | --- |
| Arachidonic acid | y=0.00255x+0.0126 | 0.9985 | 0.5-400 |
| 5-HETE | y=0.0174x-0.000940 | 0.9988 | 0.01-50 |
| 15-HETE | y=0.0340x-0.00176 | 0.9985 | 0.01-50 |
| 8,9-EET | y=0.0121x-0.00571 | 0.9969 | 0.01-50 |
| 14,15-DHET | y=0.0493x+0.000120 | 0.9967 | 0.01-50 |
| PGE_2_ | y=0.0144x-0.00438 | 0.9970 | 0.5-400 |
| PGD_2_ | y=0.0131x-0.00245 | 0.9996 | 0.5-400 |
