# Supplementary material for: Abnormal arachidonic acid metabolic network may reduce sperm motility via P38 MAPK
Source: Open Biol. 2019 Apr 24;9(4):180091. doi: 10.1098/rsob.180091 (PMC6501647; doi:10.1098/rsob.180091)
Supplement: Supplementary Table 5 [file rsob180091supp6.doc]

**Open Biology**

**Abnormal arachidonic acid metabolic network may reduce sperm motility via P38 MAPK**

Lisha Yu^1^, Xiaojing Yang^1^, Bo Ma^1^, Hanjie Ying^2^, Xuejun Shang^3,***^ , Bingfang He^1,**^, Qi Zhang^1,*^

**Supplementary Table 5.** Summary of the intra-day and inter-day precision and accuracy of representative AA metabolites. (n = 6)

| **Compound** | **Nominal concentration (ng/mL)** | **Intra-day** | | | **Inter-day** |
| --- | --- | --- | --- | --- | --- |
|  |  | **Measured concentration (ng/mL)** | **Precision (RSD, %)** | **Accuracy (RE, %)** | **Precision (RSD, %)** |
| Arachidonic | 1 | 1.04 ± 0.12 | 11.84 | 3.71 | 8.55 |
| acid | 50 | 48.27 ± 3.23 | 7.05 | -3.46 | 2.84 |
|  | 200 | 204.91 ± 14.64 | 6.70 | 2.46 | 9.86 |
| 5-HETE | 0.2 | 0.21 ± 0.02 | 10.28 | 4.35 | 8.27 |
|  | 2.5 | 2.44 ± 0.10 | 4.41 | -2.31 | 1.78 |
|  | 20 | 18.94 ± 2.00 | 10.93 | -5.32 | 7.10 |
| 15-HETE | 0.2 | 0.19 ± 0.02 | 9.68 | -6.25 | 6.79 |
|  | 2.5 | 2.54 ± 0.13 | 4.63 | 1.41 | 7.56 |
|  | 20 | 21.40 ± 2.30 | 11.21 | 6.98 | 6.09 |
| 8,9-EET | 0.2 | 0.20 ± 0.02 | 9.40 | 1.69 | 4.05 |
|  | 2.5 | 2.45 ± 0.11 | 4.32 | -2.01 | 5.85 |
|  | 20 | 20.89 ± 2.03 | 10.16 | 4.43 | 5.24 |
| 14,15-DHET | 0.2 | 0.21 ± 0.02 | 9.90 | 6.49 | 7.06 |
|  | 2.5 | 2.62 ± 0.20 | 8.00 | 4.96 | 5.83 |
|  | 20 | 21.22 ± 2.19 | 10.79 | 6.08 | 5.39 |
| PGE_2_ | 1 | 1.02 ± 0.06 | 6.09 | 1.98 | 3.56 |
|  | 50 | 50.71 ± 2.87 | 5.31 | 1.42 | 7.72 |
|  | 200 | 207.62 ± 14.79 | 6.83 | 3.81 | 9.00 |
| PGD_2_ | 1 | 0.98 ± 0.05 | 4.83 | -1.71 | 8.08 |
|  | 50 | 50.97 ± 3.47 | 7.18 | 1.94 | 2.85 |
|  | 200 | 208.59 ± 14.48 | 6.53 | 4.29 | 9.49 |
