# Supplementary material for: Abnormal arachidonic acid metabolic network may reduce sperm motility via P38 MAPK
Source: Open Biol. 2019 Apr 24;9(4):180091. doi: 10.1098/rsob.180091 (PMC6501647; doi:10.1098/rsob.180091)
Supplement: Supplementary Table 6 [file rsob180091supp7.doc]

**Open Biology**

**Abnormal arachidonic acid metabolic network may reduce sperm motility via P38 MAPK**

Lisha Yu^1^, Xiaojing Yang^1^, Bo Ma^1^, Hanjie Ying^2^, Xuejun Shang^3,***^ , Bingfang He^1,**^, Qi Zhang^1,*^

**Supplementary Table 6.** Individual data of sperm motility included in Fig 2. (n=5)

| **Fig. 2A** | | | | **Fig. 2C** | | | | **Fig. 2E** | | | | | | |
| --- | --- | --- | --- | --- | --- | --- | --- | --- | --- | --- | --- | --- | --- | --- |
| **Control** | **20μM AA** | **40μM AA** | **60μM AA** | **Control** | **AA** | **Aniso-mycin** | **AA+**  **SB** | **Control** | **AA** | **AA+**  **SC** | **AA+**  **NS** | **AA+**  **Diclo** | **AA+**  **AA861** | **AA+**  **C26** |
| 65 | 60 | 55 | 55 | 63 | 50 | 50 | 60 | 65 | 55 | 54 | 65 | 59 | 53 | 52 |
| 60 | 62 | 58 | 48 | 59 | 49 | 45 | 59 | 50 | 43 | 52 | 59 | 49 | 47 | 61 |
| 55 | 59 | 50 | 40 | 56 | 52 | 40 | 53 | 61 | 42 | 49 | 55 | 51 | 51 | 57 |
| 63 | 53 | 48 | 45 | 55 | 46 | 34 | 54 | 63 | 39 | 57 | 48 | 53 | 51 | 53 |
| 51 | 45 | 53 | 35 | 53 | 51 | 53 | 53 | 51 | 43 | 52 | 56 | 55 | 59 | 53 |
