# Supplementary material for: Abnormal arachidonic acid metabolic network may reduce sperm motility via P38 MAPK
Source: Open Biol. 2019 Apr 24;9(4):180091. doi: 10.1098/rsob.180091 (PMC6501647; doi:10.1098/rsob.180091)
Supplement: Supplementary Table 7 [file rsob180091supp8.doc]

**Open Biology**

**Abnormal arachidonic acid metabolic network may reduce sperm motility via P38 MAPK**

Lisha Yu^1^, Xiaojing Yang^1^, Bo Ma^1^, Hanjie Ying^2^, Xuejun Shang^3,***^ , Bingfang He^1,**^, Qi Zhang^1,*^

**Supplementary Table 7.** Intensity of western blot results included in Fig 2. (n=3)

Fig. 2B

|  |  | **Control** | **20μM AA** | **40μM AA** | **60μM AA** |
| --- | --- | --- | --- | --- | --- |
| 1st | p-P38 | 48093 | 51501 | 60849 | 67759 |
|  | P38 | 88056 | 84387 | 80411 | 78729 |
|  | GAPDH | 61856 | 53150 | 52010 | 54453 |
| 2nd | p-P38 | 32387 | 53507 | 63202 | 72941 |
|  | P38 | 58820 | 64329 | 64757 | 65520 |
|  | GAPDH | 67881 | 74431 | 66444 | 67441 |
| 3rd | p-P38 | 38757 | 46010 | 63002 | 82757 |
|  | P38 | 85801 | 85367 | 89948 | 88576 |
|  | GAPDH | 71349 | 72169 | 74213 | 80127 |

|  |  | **Control** | **20μM AA** | **40μM AA** | **60μM AA** |
| --- | --- | --- | --- | --- | --- |
| 1st | p-ERK (Thr202) | 44975 | 49391 | 45947 | 46260 |
|  | p-ERK (Thr204) | 47447 | 47541 | 44685 | 45000 |
|  | ERK (Thr202) | 56335 | 61528 | 57441 | 54026 |
|  | ERK (Thr204) | 67610 | 72671 | 69068 | 65264 |
|  | GAPDH | 69526 | 72338 | 70470 | 66210 |
| 2nd | p-ERK (Thr202) | 41164 | 40608 | 38069 | 40341 |
|  | p-ERK (Thr204) | 38395 | 38573 | 39615 | 39017 |
|  | ERK (Thr202) | 45550 | 48049 | 50384 | 47878 |
|  | ERK (Thr204) | 36055 | 37556 | 40274 | 37804 |
|  | GAPDH | 61329 | 56414 | 55791 | 63051 |
| 3rd | p-ERK (Thr202) | 42364 | 44425 | 45936 | 41352 |
|  | p-ERK (Thr204) | 31854 | 33223 | 33467 | 31545 |
|  | ERK (Thr202) | 43082 | 41309 | 42278 | 38742 |
|  | ERK (Thr204) | 35724 | 32575 | 34135 | 32165 |
|  | GAPDH | 70593 | 72432 | 74602 | 71610 |

|  |  | **Control** | **20μM AA** | **40μM AA** | **60μM AA** |
| --- | --- | --- | --- | --- | --- |
| 1st | p-JNK (Thr183) | 50829 | 54415 | 55200 | 50136 |
|  | p-JNK (Thr185) | 40502 | 42132 | 43840 | 41049 |
|  | JNK (Thr202) | 56562 | 60885 | 60407 | 58932 |
|  | JNK (Thr204) | 58657 | 62218 | 63179 | 60955 |
|  | GAPDH | 57871 | 60109 | 61997 | 58976 |
| 2nd | p-JNK (Thr183) | 63458 | 62290 | 65692 | 62569 |
|  | p-JNK (Thr185) | 47789 | 46424 | 50685 | 45967 |
|  | JNK (Thr202) | 64830 | 62835 | 58544 | 59019 |
|  | JNK (Thr204) | 49473 | 49158 | 49562 | 47949 |
|  | GAPDH | 88466 | 93175 | 90075 | 93414 |
| 3rd | p-JNK (Thr183) | 58750 | 58908 | 60003 | 61246 |
|  | p-JNK (Thr185) | 41703 | 41733 | 43342 | 44153 |
|  | JNK (Thr202) | 59357 | 57247 | 61858 | 58543 |
|  | JNK (Thr204) | 50267 | 48570 | 50383 | 47529 |
|  | GAPDH | 87890 | 82279 | 84347 | 80396 |

Fig. 2D

|  |  | **Control** | **AA** | **Anisomycin** | **AA+SB** |
| --- | --- | --- | --- | --- | --- |
| 1st | p-P38 | 29145 | 61354 | 50915 | 33953 |
|  | P38 | 48820 | 66673 | 71429 | 54342 |
|  | GAPDH | 64478 | 71620 | 67986 | 60666 |
| 2nd | p-P38 | 47639 | 79051 | 69639 | 50477 |
|  | P38 | 76943 | 76623 | 73179 | 74269 |
|  | GAPDH | 74757 | 70070 | 72434 | 67644 |
| 3rd | p-P38 | 28615 | 53885 | 43071 | 31103 |
|  | P38 | 56906 | 60283 | 55901 | 52663 |
|  | GAPDH | 63970 | 62181 | 60201 | 60640 |

Fig. 2F

|  |  | **Control** | **AA** | **AA+SC** | **AA+NS** | **AA+Diclo** | **AA+AA861** | **AA+C26** |
| --- | --- | --- | --- | --- | --- | --- | --- | --- |
| 1st | p-P38 | 43040 | 79175 | 56765 | 56856 | 55455 | 56082 | 55912 |
|  | P38 | 61987 | 60131 | 61151 | 60608 | 60716 | 57334 | 58767 |
|  | GAPDH | 65972 | 64355 | 67406 | 64136 | 63537 | 64548 | 63115 |
| 2nd | p-P38 | 41496 | 75788 | 49028 | 49219 | 46697 | 51385 | 48806 |
|  | P38 | 75130 | 76965 | 73749 | 73633 | 74070 | 78515 | 74893 |
|  | GAPDH | 77690 | 77040 | 79395 | 74494 | 75856 | 74479 | 72840 |
| 3rd | p-P38 | 43156 | 83003 | 50592 | 49922 | 44789 | 46162 | 45581 |
|  | P38 | 65131 | 66480 | 66268 | 65569 | 63532 | 60875 | 60956 |
|  | GAPDH | 76538 | 77583 | 76085 | 77706 | 73371 | 72691 | 71901 |
